# Conventional *Vs* Digital Impressions: Acceptability, Treatment Comfort and Stress Among Young Orthodontic Patients

**DOI:** 10.2174/1874210601812010118

**Published:** 2018-01-31

**Authors:** Alessandro Mangano, Matteo Beretta, Giuseppe Luongo, Carlo Mangano, Francesco Mangano

**Affiliations:** 1 Private practice, Piazza Trento 4, 22015 Gravedona ed Uniti, Como, Italy; 2 Private practice, Casale Monferrato, Alessandria, Italy; 3 Department of Oral and Maxillofacial Surgery, University of Naples, Naples, Italy; 4 Department of Dental Sciences, S. Raffaele University, Milan, Italy; 5 Department of Surgical and Morphological Science, Dental School, University of Varese, Varese, Italy

**Keywords:** Digital impressions, Intraoral scanners, Patients’ preferences, Treatment comfort, Comfortable impression technique, Alginate impressions

## Abstract

**Objective:**

The objective of the present study was to compare patients’ acceptability, comfort and stress with conventional and digital impressions.

**Materials and Methods:**

Thirty young orthodontic patients (15 males and 15 females) who had no previous experience of impressions were enrolled in this study. Conventional impressions for orthodontic study models of the dental arches were taken using an alginate impression material (Hydrogum^®^, Zhermack Spa, Badia Polesine, Rovigo, Italy). Fifteen days later, digital impressions of both arches were acquired using an intraoral scanner (CS3600^®^, Carestream Dental, Rochester, NY, USA). Immediately after impression taking, patients’ acceptability, comfort and stress were measured using two questionnaires and the State anxiety scale.

**Results:**

Data showed no difference in terms of anxiety and stress; however, patients preferred the use of digital impressions systems instead of conventional impression techniques. Alginate impressions resulted as fast as digital impressions.

**Conclusions:**

Digital impressions resulted the most accepted and comfortable impression technique in young orthodontic patients, when compared to conventional techniques.

## 1 INTRODUCTION

A pronounced gag reflex may be a potential problem for the acceptance and delivery of dental treatments. Even if there is the availability of a range of management strategies, even simple dental procedures are not accepted by some patients. Gagging problems are not uncommon in daily dental practice and the exact prevalence is unknown. This can have an important impact on treatment plan and dental treatment outcome [1].

The use of intraoral scanners for study models has increased dramatically among orthodontists. Digital scanners are capable to obtain high quality impressions and to reduce several problems like the gag reflex [2].

Intraoral scanning systems were introduced in dentistry in the mid 1980s. It was forecasted that the major part of the dentists in the U.S. and Europe would be using intraoral scanners for taking impressions in the next decades [3].

Digital impressions can offer a variety of advantages such as reduced patient discomfort, time-efficiency, simplified clinical procedures, and ability of capturing and storing highly accurate information (the 3D virtual models of patients) without pouring stone casts. The possibility of avoiding pouring stone casts can save space and time in the clinic. Further advantages of the digital impressions and scanning systems are the possibility to easily transfer digital data to the dental technician, *via* email, avoiding impression shipping to the laboratory: this results in a better communication with the laboratory [4]. The dental technician can immediately visualize tooth preparations (or the position of implant scanbodies), and this guarantees a better communication.

Digital dentistry is transforming the relationship between dentist and dental laboratory. As a part of this trend, intraoral scanners are playing a pivotal role to this changing relationship [5]. In the last few years, several studies have dealt with IOS and their use in different fields of dentistry [6, 7]. However, only a few studies have compared the patient preference and comfort with digital and conventional impressions [6-8] in particular among young patients [8].

Hence, the aim of the present study was to evaluate the patients’ preferences and attitudes towards the digital impression technique compared to the conventional impression technique, in young orthodontic patients. The null hypothesis was that there is no difference in patients’ preference and treatment comfort between the conventional and digital impression techniques.

## 2 MATERIALS AND METHODS

### 2.1 Study Design, Sample and Clinical Scenario

The study population consisted of 30 subjects (15 males and 15 females) referring to one private practice (Studio Odontoiatrico Mangano, Gravedona, Como, Italy). The age ranged from 7 to 16 years (mean age 11 years ± 4 months). The subjects had no previous experience of conventional or digital impressions. The subjects and parents or legal representative were informed about the clinical procedures and of possible risks and benefits, a signed consent form was obtained for all patients. All the subjects underwent impression taking in order to obtain orthodontic study models. The inclusion and exclusion criteria are summarized in Table **1**. The study protocol was conducted in accordance with the Helsinki Declaration of 1975, as revised in 2007. A priori sample size calculation was performed with α = 0.05 and a power set at 80%.

### 2.2 Conventional Impressions

The proper tray for maxillary and mandibular arches was selected by one operator (FM). The conventional impressions of both arches were made using an alginate impression material (Hydrogum, Zhermack Spa, Badia Polesine, Rovigo, Italy). The impression material was prepared according to manufacturer’s instructions and recommendations. The acceptability and perceptions of subjects were recorded immediately after the procedure using a standardized questionnaire. Patients’ attitude and discomfort was tested immediately after the impression using a VAS (Visual analogue scale). The perceived source of stress was assessed using a State anxiety scale.

### 2.3 Digital Impressions

**Fig. (1):**
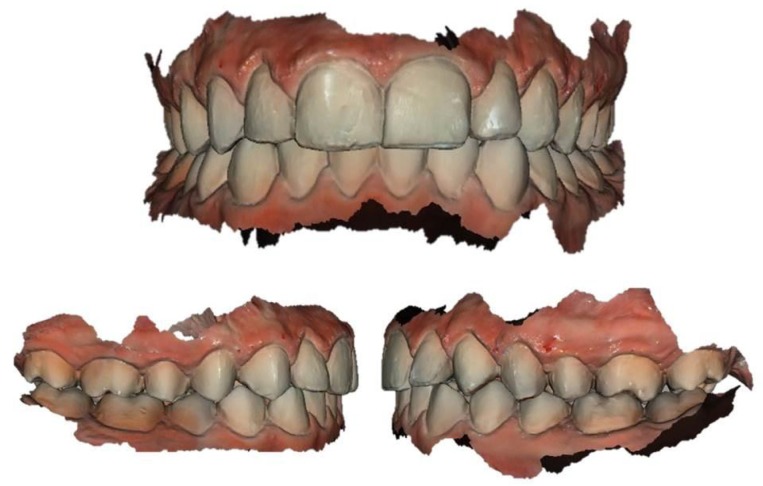
Digital impression of the dental arches of a young orthodontic patient with a definitive dentition.

**Fig. (2):**
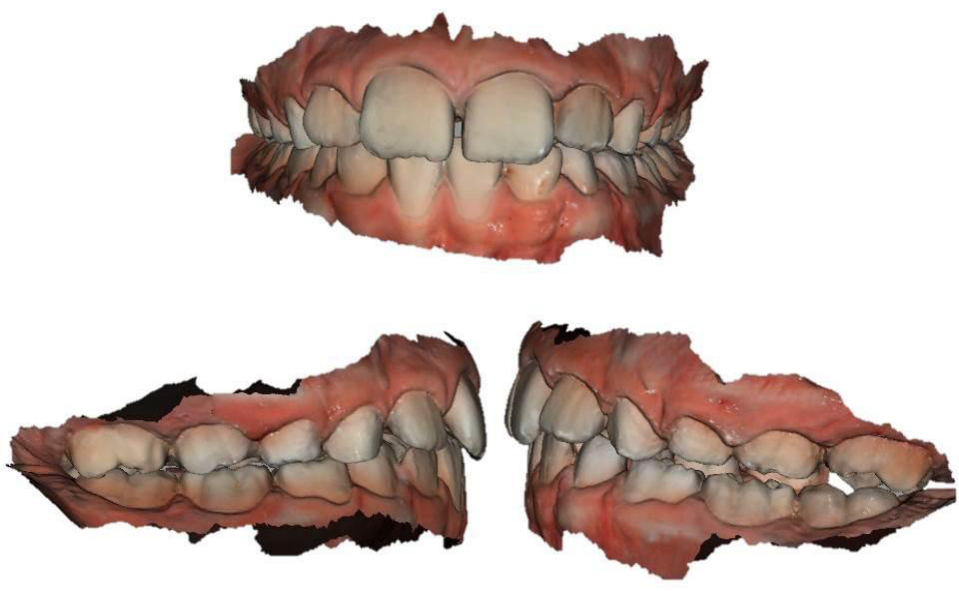
Frontal, right and left view of the dental arches of a young orthodontic patient with a mixed dentition.

A digital impression of both arches (Figs. **1** and **2**) was obtained 15 days after the conventional impressions appointment. The digital impressions were performed using an intraoral scanner (CS3600^®^, Carestream, Rochester, NY, USA). The digital data of both arches were recorded according to manufacturer’s instructions by the same operator (FM). The acceptability and perceptions of subjects were recorded immediately after the procedure using a standardized questionnaire. Patients’ attitude and discomfort was tested immediately after the impression using a VAS (Visual Analogue Scale). The perceived source of stress was assessed using a State anxiety scale (S-scale).

### 2.4 Statistical Analysis

The acceptability and perceptions of the subjects on both impression techniques were assessed with a self-administrated questionnaire using a Visual Analog Scale (VAS) ranging from 0 to 100. The data were analyzed statistically applying the Wilcoxon Signed-Rank Test, the level for statistical significance was set at *p* = 0.05, using the *SPSS* 15.0 statistical software (*SPSS* Inc., Chicago, IL, USA). The subjects’ preferences for the impression techniques were assessed with a 9-item comparative questionnaire [7]. Descriptive statistical analysis using the *SPSS* 15.0 statistical software (*SPSS* Inc., Chicago, IL, USA) was used to evaluate the distribution of the answers. The perceived source of stress was evaluated using a State anxiety scale and statistically analyzed by Wilcoxon Signed-Rank Test (*p*=0.05).

## 3 RESULTS

### 3.1 Patients’ Acceptability and Perceptions

The evaluation scores of acceptability and perceptions by the patients are summarized in Table **2**. The subjects’ level of stress was evaluated by State anxiety scale. The mean scores of this test were not significant (*p*>0.05). The mean scores of the subjects perceptions criteria were significantly different (*p*<0.001) except for overall time impression. The conventional impression technique resulted to be slighlty faster in terms of time. The digital impression technique was the most accepted by patients and all subjects preferred this technique (*p*<0.001). The 9-item questionnaire scores are summarized in Table **3**.

## 4 DISCUSSION

In this clinical trial, according to the clinical scenario developed, the digital impression technique was more accepted by patients in terms of comfort and overall acceptance. Thus, the null hypothesis was rejected. The study sample obtained was standardized and homogenized by including subjects who had no previous experience with any kind impression techniques.

To investigate the acceptance and stress perceived by the patients, homogenizing the study population is a wide-accepted clinical research method in order to optimize objectivity and minimize bias. This approach is important when it comes to objectively report patients’ acceptance avoiding report of bias of patients who had previous experience with the dental impression techniques. In this present clinical trial we mainly focused in evaluating the stress perceptions and overall procedure acceptability of two impression techniques. The results showed how statistically significant differences were found in overall time impression. This can be attributable to the fast setting time of the alginate material [9].

In terms of patients’ acceptability and comfort all the parameters investigated resulted to be statistically significant when comparing the use of digital impression systems to conventional impression techniques.

These findings are in contrast with those of Grünheid *et al*. [2], who observed that patients preferred the conventional impression technique because of dimension of scanner’s tip. This problem has been overcome thanks to the even more narrow dimensions of intraoral scanners’ wands [10].

Our results showed that the 100% of the sample preferred the digital impression. This can be attributable to a thinner scan design [11].

Two recent trials [7, 12] showed the same trend of patients’ preference reporting that intraoral scanner are more accepted than traditional impression techniques.

One of the limitations of this study is that we analyzed only one intraoral scanner system, other digital systems and their workflows could lead to different results. Therefore, it cannot be generalized that all digital impression systems are capable to give the same results.

Our data revealed that there were statistically significant differences both in gag reflex and breathing difficulty. Gagging problems are encountered in daily dental practice [13]. The occurrence of nausea, while performing dental procedures is a major problem to providing good-quality dental treatment, especially when it is necessary to take impressions of the dental arches [14]. Moreover, the treatment plan could be compromised and limited by the need to limit the impact of the gag reflex. Furthermore, some patients may require more invasive levels of intervention such as anesthesia (local or general) or conscious sedation [14]. Data about the exact prevalence of the gag reflex in the general population are not available, but it undoubtedly affects many patients [15]. According to our data it can be assumed that IOS systems could easily overcome these problems.

To the authors’ knowledge, this is the second clinical trial involving adolescents in evaluating acceptability of digital impression technique. In fact, in a previous similar study, Burhardt *et al*. [8]

Assessed preferences for impression techniques in young orthodontic patients receiving alginate and two different digital impressions. In total, the authors selected 38 subjects (aged between 10 - 17 years) requiring impressions for orthodontic treatment: these were randomly allocated to three groups, that differed in the order that an alginate impressions and two different intraoral scanning procedures (Omnicam, Sirona and Lava COS, 3M) were administered [8]. After each procedure, the patients were asked to score their perceptions on a 5-point Likert scale for gag reflex, queasiness, difficulty to breathe, uncomfortable feeling, perception of the scanning time, state of anxiety, and use of a powder, and to select the preferred impression system [8]. Chairside time was also registered. More queasiness (*P* = 0.00) and discomfort (*P* = 0.02) during alginate impression taking of the maxilla were perceived compared with the scans with the Omnicam [8]. There were no significant differences in perceptions between the alginate impressions and the Lava COS and between the two scanners. Chairside times for the alginate impressions (9.7 ± 1.8 minutes) and the Omnicam (10.7 ± 1.8 minutes) were significantly lower (*P* <0.001) than for the Lava COS (17.8 ± 4.0 minutes). Digital impressions were favored by 51% of the subjects, whereas 29% chose alginate impressions, and 20% had no preference. Regardless of the significant differences in the registered times among the 3 impression-taking methods, the distributions of the Likert scores of time perception and maximal mouth opening were similar in all three groups [8]. The authors therefore concluded that young orthodontic patients preferred the digital impression techniques over the alginate method, although alginate impressions required the shortest chairside time [8].

Considering the limited number of studies available in the present literature, still it is not possible to state if there are age-related differences in patients’ acceptability and stress with different kind of impressions. Further studies should analyze whether or not there could be age-related differences among patients’ perceptions. However, our data showed that digital impression technique resulted to be more patient-friendly than the conventional impression technique. Further studies with wider sample and comparing different age groups should be performed in order to deeply investigate those aspects.

## CONCLUSION

Within the limits of the present investigation, the following conclusions can be drawn:

- The overall patients’ acceptance of digital impression techniques was significantly higher than that of conventional impression techniques;

- Intraoral scanner scored a better value in terms of comfort, gag reflex and breathing difficulty;

- No significant differences were found between the two techniques in terms of stress (State anxiety scale).

- Alginate impressions resulted to be slightly faster than digital impressions.

## Figures and Tables

**Table 1:** Inclusion and exclusion criteria.

**Inclusion Criteria**	**Exclusion Criteria**
Good oral health	Previous experiences with impressions
Good oral hygiene	History of orthodontic treatments
No periodontal disease	Presence of prosthetic restorations

**Table 2:** Patients’ acceptability, feelings and stress perceived. *p<0.05.

**Evaluation (VAS)**	**Conventional (Mean±SD)**	**Digital (Mean±SD)**	***P-*value**
Overall discomfort	52,50±9,26	90,50±6,20	<0,001*
Overall time impression	51,40±8,65	76,50±9,39	<0,001*
Smell/Voice	55,83±8,31	75,63±22,21	<0,001*
Taste/Heat	54,67±7,76	89,00±7,24	<0,001*
GAG reflex	46,33±7,64	90,00±6,29	<0,001*
Discomfort during mouth was open	41,67±7,69	86,83±7,36	<0,001*
TMJ discomfort	44,50±7,58	88,00±7,83	<0,001*
Breathing impairment	45,50±8,23	87,50±10,23	<0,001*
Teeth and periodontal sensitivity	58,33±8,44	91,17±6,90	<0,001*
Total evaluation score	477,50±36,80	775,13±43,01	<0,001*
***Level of Source of Stress***	–	–	–
Score of S-scale	24,67±11,36	21,00±10,61	>0,05

**Table 3:** Patients’ preferences about impression techniques.

Preferences	Conventional	Digital
Which impression technique do you prefer in case of one more time for impression procedure?	0%	100%
Which impression technique is more comfortable from point of comparison of two impression procedure?	0%	100%
Which impression technique do you suggest in case of a friends’ need for impression making?	0%	100%
Which impression technique do you prefer from point of time involved with impression procedure?	0%	100%
Which impression technique do you prefer from point of feeling taste/smell or voice/heat during impression procedure?	0%	100%
Which impression technique do you prefer from point of the size of the intraoral scanner/impression tray used in your mouth during impression procedure?	0%	100%
Which impression technique do you prefer from point of having tooth/gingival sensitivity during impression procedure?	0%	100%
Which impression technique do you prefer from point of having difficulty in breathing during impression procedure?	0%	100%
Which impression technique do you prefer from point of having gagging reflex during impression procedure?	0%	100%

## References

[1] Zotelli V.L., Grillo C.M., de Sousa Mda.L. (2014). Nausea control by needling at acupuncture point Neiguan (PC6) during an intraoral impression-taking procedure.. J. Acupunct. Meridian Stud..

[2] Grünheid T., McCarthy S.D., Larson B.E. (2014). Clinical use of a direct chairside oral scanner: An assessment of accuracy, time, and patient acceptance.. Am. J. Orthod. Dentofacial Orthop..

[3] Imburgia M., Logozzo S., Hauschild U., Veronesi G., Mangano C., Mangano F.G. (2017). Accuracy of four intraoral scanners in oral implantology: A comparative *in vitro* study.. BMC Oral Health.

[4] Grauer D., Proffit W.R. (2011). Accuracy in tooth positioning with a fully customized lingual orthodontic appliance.. Am. J. Orthod. Dentofacial Orthop..

[5] Christensen G.J. (2009). Impressions are changing: deciding on conventional, digital or digital plus in-office milling.. J. Am. Dent. Assoc..

[6] Joda T., Brägger U. (2015). Time-efficiency analysis comparing digital and conventional workflows for implant crowns: A prospective clinical crossover trial.. Int. J. Oral Maxillofac. Implants.

[7] Yuzbasioglu E., Kurt H., Turunc R., Bilir H. (2014). Comparison of digital and conventional impression techniques: Evaluation of patients’ perception, treatment comfort, effectiveness and clinical outcomes.. BMC Oral Health.

[8] Burhardt L., Livas C., Kerdijk W., van der Meer W.J., Ren Y. (2016). Treatment comfort, time perception, and preference for conventional and digital impression techniques: A comparative study in young patients.. Am. J. Orthod. Dentofacial Orthop..

[9] Mangano F.G., Veronesi G., Hauschild U., Mijiritsky E., Mangano C. (2016). Trueness and precision of four intraoral scanners in oral implantology: A comparative *in vitro* study.. PLoS One.

[10] Ender A., Mehl A. (2013). Influence of scanning strategies on the accuracy of digital intraoral scanning systems.. Int. J. Comput. Dent..

[11] van der Meer W.J., Vissink A., Ren Y. (2016). Full 3-dimensional digital workflow for multicomponent dental appliances: A proof of concept.. J. Am. Dent. Assoc..

[12] Wismeijer D., Mans R., van Genuchten M., Reijers H.A. (2014). Patients’ preferences when comparing analogue implant impressions using a polyether impression material versus digital impressions (Intraoral Scan) of dental implants.. Clin. Oral Implants Res..

[13] Kaakko T., Horn M.T., Weinstein P., Kaufman E., Leggott P., Coldwell S.E. (2003). The influence of sequence of impressions on children’s anxiety and discomfort.. Pediatr. Dent..

[14] Dickinson C.M., Fiske J. (2005). A review of gagging problems in dentistry: I. Aetiology and classification.. Dent. Update.

[15] Ramsay D.S., Weinstein P., Milgrom P., Getz T. (1987). Problematic gagging: Principles of treatment.. J. Am. Dent. Assoc..

